# The study of the effect of virtual reality technology combined with sports games on improving cognitive function in patients with brain injury: a meta analysis of randomized controlled trials

**DOI:** 10.3389/fneur.2025.1579909

**Published:** 2025-05-13

**Authors:** Yuze Zhang, Haojie Li, Hong Xiao

**Affiliations:** ^1^Department of Martial Arts and Ethnic Traditions, Henan Sport University, Zhengzhou, China; ^2^School of Physical Education, Yunnan Normal University, Yunnan, China; ^3^School of Exercise and Health, Shanghai University of Sport, Shanghai, China

**Keywords:** virtual game technology, brain-injured patients, cognitive function, RCT, novel rehabilitation modality

## Abstract

**Background:**

Brain injury is a major public health issue causing cognitive impairment. Key types include traumatic, ischemic, neurological, infectious, metabolic injuries, and stroke. As populations age, brain injury rates rise, making effective cognitive rehabilitation methods increasingly urgent. Virtual reality sports games, blending immersion and training, offer a new rehab solution.

**Methods:**

Firstly, we registered in the International Prospective Systematic Review Registry (PROSPERO) website. A total of 12 randomized controlled trials were included in this Meta-analysis. Data were analyzed by Meta-analysis using the random effects model in State statistical software. The primary outcome indicator of the study was cognitive function.

**Results:**

This study included 12 RCTs with 540 participants to evaluate the impact of virtual reality exercise on cognitive function in brain-injured patients. The analysis revealed significant cognitive improvement with an SMD of 0.88 (95% CI: 0.59, 1.17), *p* = 0.019, and I^2^ = 51.9% using a random effects model. Sensitivity analysis confirmed robust findings with no significant single study effects. Symmetric funnel plots indicated no publication bias. These results support virtual reality as an effective cognitive intervention for brain-injured patients.

**Conclusion:**

Virtual reality (VR) sports games significantly enhanced cognitive function, coordination, and reaction speed in brain-injured patients, thereby boosting their learning motivation and engagement.

**Systematic Review Registration:**

https://www.crd.york.ac.uk/PROSPERO/, identifier CRD42024581533.

## 1 Introduction

Brain injury is a global public health issue with a rising incidence annually. The World Health Organization reports that it results in millions of deaths and disabilities each year (1). Individuals with brain injury often encounter severe consequences, such as diminished physical function, cognitive deficits, and emotional disturbances. The aging population further exacerbates this issue, as the incidence of brain injury is gradually increasing, making associated clinical problems more pronounced (2, 3). These injuries can lead to a range of symptoms, including memory loss, inattention, executive dysfunction, and reduced social interaction, which not only affect patients’ daily lives but also place a significant burden on families and society (4). Stroke, in particular, is a significant type of brain injury with substantial clinical implications. Research indicates that stroke patients often experience marked cognitive decline, especially in attention, memory, and executive function, following the onset of the disease (5). This underscores the importance of research focused on cognitive recovery in brain-injured patients. Cognitive function refers to an individual’s capacity to acquire, process, store, and apply information. Current research on brain-injured patients primarily focuses on deficits in cognitive and executive functions (6). Patients with brain injury have different degrees of impairment in working memory, attention, executive function, decision-making ability, language, visuospatial ability and emotional regulation (7–10). Current studies concentrate on specific cognitive domains in brain-injured patients, lacking a comprehensive and systematic evaluation of cognitive function across different types of brain injuries. Moreover, traditional intervention methods often fail to meet patients’ individual needs and lack effective tools to monitor rehabilitation progress (11). Therefore, the exploration of virtual reality sports game-based interventions is essential for identifying effective rehabilitation methods to enhance cognitive functions in brain-injured patients. This approach not only addresses the limitations of current interventions but also provides a robust scientific basis and practical guidance for patient rehabilitation.

Previous studies have focused on the effects of various exercise modalities on cognitive function in brain-injured patients, including traditional physical exercise, cognitive training, and music therapy (12–15). However, these interventions are still insufficient in improving participation, fun and reducing side effects (16). With the development of artificial intelligence technology, although some studies have begun to attempt to incorporate technological means for intervention, existing interventions still lack personalization and interactivity (17). Therefore, it is necessary to explore newer interventions to improve intervention effectiveness and patient engagement.

Virtual reality sports gaming technology is an emerging intervention that integrates elements of sports and gaming, utilizing virtual reality to enhance the enjoyment and sustainability of training (18). Virtual reality technology enables patients to perform sports training in a safe environment by creating immersive virtual environments. The use of this technology not only improves patient engagement, but also offers the possibility of personalized treatment (19). Virtual reality gaming technology can improve the cognitive function of brain-injured patients by engaging multiple cognitive domains through interactive and immersive experiences. Games such as “Beat Saber,” “VR Boxing,” and “Dance Central VR” require sustained attention, rapid decision-making, and coordination of physical movements with visual and auditory stimuli. These activities challenge memory, spatial awareness, and executive functions, promoting neuroplasticity and cognitive recovery. The engaging nature of these games also enhances motivation and adherence to rehabilitation protocols. In addition, sensor-based devices (e.g., motion capture sensors, accelerometers, and gyroscopes) are widely used in these virtual motion technologies. Virtual games capture movements in real time, provide instant feedback, and increase patient engagement, motor skills, and attention (20).

Studies have shown that virtual reality sports games have the potential to improve cognitive function in brain-injured patients in several ways (21). First, the interactive and immersive experience provided by virtual reality can stimulate patients’ interest and motivation and promote active participation in sports (22). Second, this technology can enhance learning through multisensory stimulation and immediate feedback, thereby promoting neuroplasticity and improving cognitive performance (4). Third, the adjustability of virtual reality environments allows therapists to personalize and optimize training regimens according to patients’ specific needs and abilities (23). Therefore, the intervention based on virtual reality sports games can not only enrich the means of rehabilitation for brain-injured patients, but also effectively enhance the recovery of their cognitive functions (24).

This study innovatively pioneers the use of virtual reality sports game technology to improve brain-injured patients’ cognitive function. We’ll conduct a randomized controlled trial to systematically evaluate its effects, providing a scientific basis for clinical practice. Unlike traditional methods, it enhances patients’ participation and enjoyment and boosts training effectiveness via diverse exercises and interactive experiences (25). By comprehensively analyzing existing research, we aim to uncover the underlying mechanisms, offering theoretical and empirical support for future practice. The paper is structured as follows: Chapter 2 presents research methods and data processing, Chapter 3 details data analysis and results, Chapter 4 discusses findings, and Chapter 5 summarizes and suggests future research directions.

## 2 Materials and methods

### 2.1 Protocol and registration

The PRISMA (Preferred Reporting Items for Systematic Reviews and Meta-Analyses) statement provides a set of comprehensive guidelines aimed at enhancing the quality of systematic reviews and meta-analyses. These guidelines encompass various stages of the review process, including literature searching, study selection criteria, data extraction, and the presentation of results. The primary goal of PRISMA is to ensure transparency and reproducibility in research practices, thereby enhancing the reliability and credibility of findings.

Adhering to the PRISMA framework requires a thorough and systematic approach to literature searching, along with the establishment of clear inclusion and exclusion criteria to ensure the studies selected are aligned with the research objectives. During the selection phase, studies that meet these criteria are identified, and the rationale for inclusion or exclusion is meticulously documented. This typically involves an initial screening, followed by a full-text assessment and final inclusion, all of which help maintain consistency and rigor across the included studies

### 2.2 Sources of information

Searches were performed in WOS, PubMed, Embase, Cochrane, EBSCO, CNKI and other databases up to October 2024. The search results were imported into Endnote.

### 2.3 Search strategy

**Figure 1:**
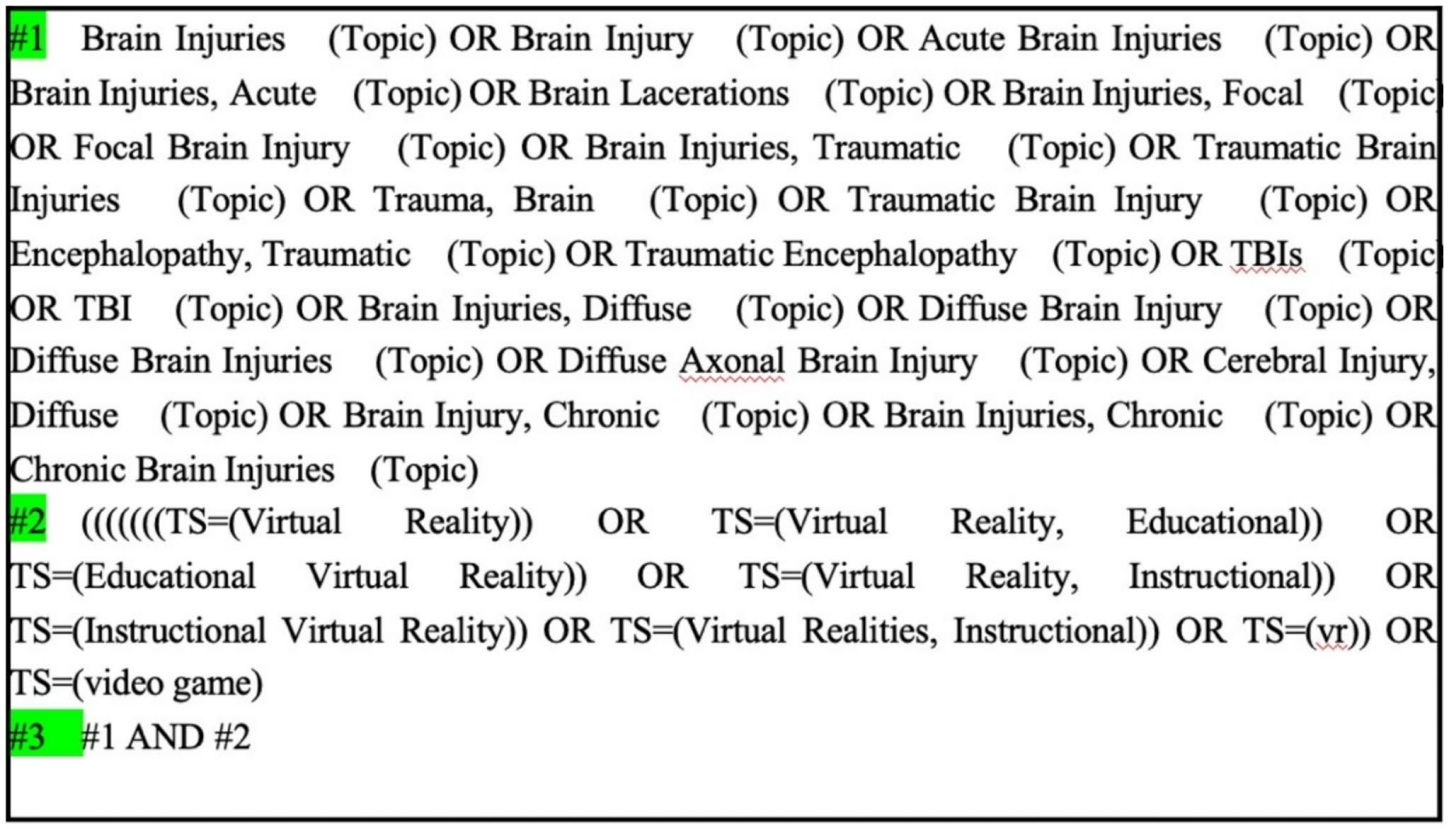
Cochrane search strategy.

The literature search was conducted with a language restriction to English and Chinese, and encompassed all relevant studies published up to April 2025. The selection of the study was independently completed by two researchers (YZ and HL). As depicted in Figure 1, which outlines the detailed search protocol from Cochrane, the following key terms were employed in identifying studies focused on the effects of virtual reality motion gaming on individuals diagnosed with brain injury (a) Virtual Reality, Virtual Reality Systems, Educational Virtual Realities, Educational VR, or Video Games, and (b) Brain Injuries, Focal Brain Injury, Traumatic Brain Injuries (TBI), Traumatic Encephalopathy, Diffuse Brain Injury, or Chronic Brain Injuries.

### 2.4 Inclusion and exclusion criteria

In this study, the inclusion criteria were defined according to the PICOS framework, which includes Population, Intervention, Comparison, Outcome, and Study Design. Specifically, the inclusion criteria for the literature review were as follows: (1) Participants were individuals with brain injuries, regardless of gender; (2) The intervention involved virtual reality motion gaming technology, such as computer-based games or programming; (3) The study design required a comparison between the intervention group and a control group; (4) Outcome measures had to assess cognitive functioning in patients with brain injuries; (5) Only randomized controlled trials (RCTs) were considered to ensure the study’s methodological rigor; (6) Studies were limited to those published in English or Chinese.

Conversely, the exclusion criteria were: (1) Studies that were not randomized controlled trials; (2) Research that did not involve patients with brain injuries; (3) Studies that did not report clear statistical data, including means and standard deviations; (4) Studies for which the full text was unavailable; (5) Research where the intervention was unrelated to virtual reality motion gaming technology; and (6) Studies published in languages other than English or Chinese.

### 2.5 Data extraction and synthesis

The process of data extraction comprised three key stages: 1. Study Selection. A comprehensive literature search was conducted across multiple databases to ensure all relevant studies were identified. Following this, precise inclusion and exclusion criteria were established to determine the studies to be considered for inclusion in the analysis and those to be excluded. The collected study was screened by two researchers (YZ, HL), and cross-checked their results. If the results were consistent, the study was included; if there was a disagreement, the third researcher (HX) was consulted, and the study was finally included after discussion. 2. Data Extraction. The extracted data encompassed various study details, including the names of authors, publication year, sample size, source of the study, participant demographics, characteristics of the intervention, assessment tools used, and the outcomes of the study. Two trained researchers (YZ, HL) independently extracted data from the included literature using standardized data extraction forms. 3. Quality Assessment of the Studies. The methodological quality of each study was evaluated using the Cochrane Risk of Bias tool. The results of these quality assessments were systematically recorded in a data extraction form for later review and analysis. Two researchers (YZ and HL) assessed the risk of bias for all eligible studies. If there was a disagreement, a third researcher (HX) participated in the discussion to reach a consensus.

Data synthesis was performed in four major steps: 1. Effect Size Calculation. Based on the type of data being analyzed, it was determined that a continuous variable was appropriate. Therefore, either the standardized mean difference (SMD) or the mean difference (MD) was chosen as the effect size metric. Effect size calculations and subsequent synthesis were conducted using Stata statistical software. 2. Heterogeneity Assessment. To evaluate the variability between studies, the I^2^ statistic was calculated. A higher I^2^ value indicates greater heterogeneity. 3. Model Selection. Given the considerable heterogeneity among the studies, a random effects model was selected for the analysis. 4. Sensitivity Analysis. A sensitivity analysis was performed on the five outcome measures to assess the influence of individual studies on the overall results and to confirm the robustness of the findings. The “data extraction and synthesis” process constitutes the foundation of meta-analysis. By adhering to a structured and methodologically rigorous approach, this process effectively consolidates and evaluates existing research, ultimately yielding more reliable and valid conclusions.

### 2.6 Outcome

The MoCA (Montreal Cognitive Assessment) is a comprehensive cognitive screening tool widely used to evaluate mild cognitive impairment (MCI) and Alzheimer’s disease (AD). It assesses multiple cognitive domains, including visuospatial function, naming, attention, executive function, visual perception, orientation, calculation, and abstract thinking. The WMS-III (Wechsler Memory Scale—Third Edition) is a comprehensive memory assessment tool that includes several subtests to evaluate immediate and delayed memory, visual memory, and verbal memory. The CBAAD (Continuous Behavioral Attention Assessment Device) is used to assess sustained attention and alertness by requiring participants to maintain a high level of attention through continuous tasks. The Simon task is a classic reaction time task used to assess response inhibition and conflict resolution abilities. The Scaled Score standardizes results from various tests, including the Wechsler scales (WTAR, WAIS-IV), the D-KEFS series of tests (trail making, color-word interference, design fluency), HVLT-R, BVMT-R, and RDS, to provide a unified scoring standard. EEG (electroencephalography) is used to assess brain activity patterns and reflect changes in neural function.

### 2.7 Assessment of research quality

The assessment of study quality is a crucial step in ensuring the validity and reliability of the results obtained from a meta-analysis. For this purpose, the Cochrane Handbook for Systematic Reviews of Interventions was selected as the primary tool for evaluating study quality. Various types of bias, including selection bias, performance bias, detection bias, attrition bias, and reporting bias, were systematically recorded for each study. The risk of bias for each included study was independently rated by two assessors, with categories of low, high, or unclear risk being assigned. The assessment process determined that studies rated with a higher number of low-risk elements were deemed to have higher methodological quality, whereas those rated with more high-risk elements were considered to have lower quality. According to the Cochrane guidelines, a study was classified as having a low risk of bias if it employed appropriate randomization methods, ensured blinding of outcome assessors, provided uniform intervention for all participants, and reported outcomes without selective omission, all of which contributed to credible findings. A classification of unclear risk was assigned when there was insufficient information available about randomization, blinding, or outcome reporting, preventing a definitive bias assessment. Studies were rated as having a high risk of bias if they lacked proper randomization, failed to blind assessors, reported incomplete results, or showed evident selection or other biases.

When evaluating the overall quality of studies, those with a low risk of bias across all domains were classified as high-quality literature. If any aspect of the study was deemed to have a high risk of bias, the entire study was categorized as low-quality. Studies that presented some, but not all, indicators of bias were classified as moderate-quality literature. Discrepancies in assessment were resolved through consensus, with a third-party reviewer involved to mediate and ensure agreement. Based on these criteria, the 12 studies included in this analysis comprised four high-quality studies, five moderate-quality studies, and three low-quality studies. Ultimately, the selected studies adhered to the required quality standards, thereby enhancing the credibility of the meta-analysis findings.

The meta-analysis in this study was performed using Stata software, applying a random-effects model for data synthesis. Effect sizes were derived based on the means and standard deviations of the outcome measures. The I^2^ statistic was utilized to assess heterogeneity among the included studies. An I^2^ value less than 50% was indicative of low heterogeneity, values between 50 and 75% represented moderate heterogeneity, and values greater than 75% suggested high heterogeneity. In cases where heterogeneity exceeded 50% (I^2^ > 50%), sensitivity analyses were conducted to explore potential sources of variability and to examine the robustness of the overall results by excluding individual studies. To assess publication bias, funnel plots were employed to visualize the relationship between sample size and effect size, allowing for an evaluation of potential publication bias.

### 2.8 Statistical analysis

In this study, a meta-analysis was conducted using the random-effects model in State software. The selection of effect sizes was as follows:

For continuous data, both the mean difference (MD) and the standardized mean difference (SMD) were utilized as effect size metrics.

Data extraction: Relevant information was systematically extracted from each individual study, including sample sizes, means, and Standard deviations, to ensure the precision and accuracy of the data used in the analysis.

Effect size calculation: The effect size for each study was computed using appropriate statistical formulas. The standard error (SE) of the effect size was also determined for each study to facilitate accurate calculations of overall effects.

Combining effect sizes: Under the random-effects model, the individual effect sizes and their respective standard errors from all studies were aggregated to compute a summary effect size. The random-effects model was chosen to account for the inherent heterogeneity among studies. Effect sizes were derived using the means and standard deviations of the outcome variables from each study. To assess heterogeneity across studies, the I^2^ statistic was employed, where values under 50% were indicative of low heterogeneity, 50–75% represented moderate heterogeneity, and values above 75% suggested high heterogeneity. When significant heterogeneity (I^2^ > 50%) was observed, a sensitivity analysis was performed to explore potential sources of this variation and evaluate the robustness of the overall findings by sequentially removing individual studies.

#### 2.8.1 Sensitivity analysis procedure

Purpose of sensitivity analysis: the aim of conducting a sensitivity analysis is to examine how the inclusion of specific studies or the choice of methodological assumptions may influence the overall results. This process tests the stability and reliability of the findings under different conditions.

#### 2.8.2 Implementation methods

Exclusion of individual studies: Studies were removed one at a time to determine the impact of each on the overall effect size. This step helps identify studies that might unduly influence the results.

Variation in effect size calculation: Different methods for calculating effect sizes were explored to assess whether the results remained consistent across various statistical approaches.

Reassessment of heterogeneity: The heterogeneity across studies was further examined by modifying the statistical model (e.g., switching from a fixed-effects to a random-effects model) to evaluate the impact of model choice on the final conclusions.

Reporting results: The results of the sensitivity analysis were presented in detail, including the methods employed and their influence on the overall effect size. This transparency allows readers to assess the robustness of the meta-analysis and understand the stability of the conclusions.

Publication bias assessment: To detect potential publication bias, funnel plots were utilized to visually examine the relationship between study sample size and effect size. This technique helps identify whether small or large studies might be disproportionately affecting the results.

## 3 Results

### 3.1 Study selection

**Figure 2:**
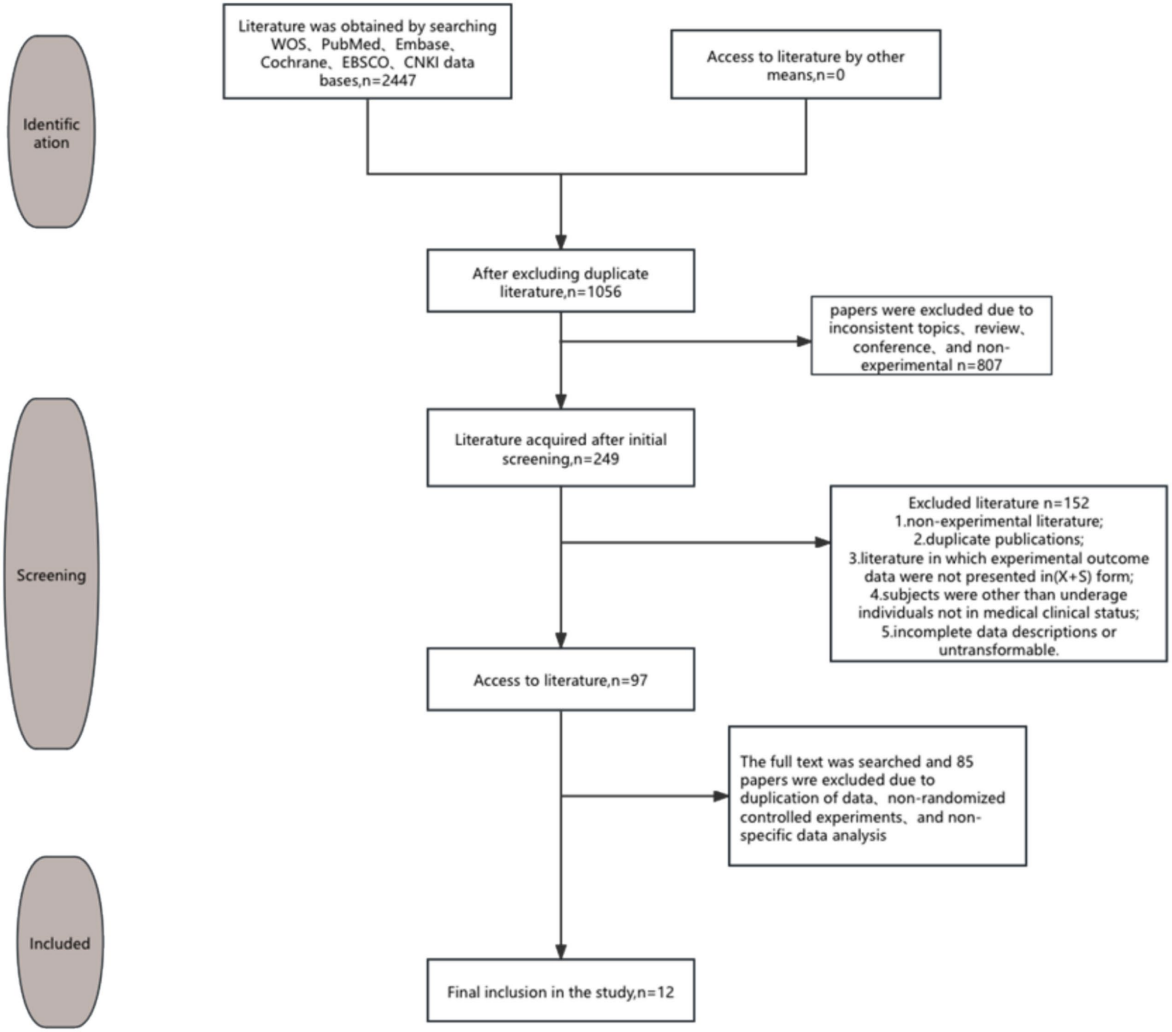
Flow chart of literature screening.

A comprehensive search was conducted across six databases—WOS, PubMed, Embase, Cochrane, EBSCO, and CNKI—yielding a total of 2,447 articles relevant to the topic of this study. Following the removal of duplicate records using Endnote21 software, 1,056 unique articles remained. After a thorough screening process, 12 randomized controlled trials (RCTs) met the inclusion criteria for this analysis. The article screening procedure, including the numbers of articles initially identified, excluded, and ultimately included, is depicted in Figure 2.

### 3.2 Basic characteristics of the included literature

A total of 12 studies were included in the present analysis, as detailed in Table 1, encompassing 540 participants from six different countries. This diverse sample provides a broad representation across various geographic regions. Among the participants, 239 were allocated to the intervention group, while the remaining participants were assigned to the control group, with a balanced gender distribution in both groups. The interventions applied in these studies were classified into three categories: (1) computer-based training, (2) motor games, and (3) virtual reality (VR) training. The intervention protocols varied significantly across the studies, reflecting the broad range of treatment strategies used.

**Table 1:** Basic features of the included studies.

Author	Year	Simple size (M/F)	Source	Age	(T/C)	Intervention cycle, frequency, and duration	Measurement tools	Information of the game
Faria et al. (31)	2020	32(16/16)	Portugal	<70	(14/18)		MoCA	Reh@City v2.0, a VR - based intervention, sets tasks in a virtual city with common places like streets, shops and parks. Patients complete ADL - related cognitive tasks such as shopping in the supermarket, picking up packages at the post office, and playing in the park.
Gamito et al. (32)	2015	20(11/9)	Portugal	mean age of 55 years (SD = 13.5)	(10/10)	4 weeks-6 weeks, 2–3 times/week, 60 min/time	WMS-III	The VR game for cognitive training includes daily - life - based attention and memory tasks like buying items, finding the way to places, and recognizing advertisements, aiming to improve patients’ related cognitive functions.
Malegiannaki et al. (33)	2024	40(20/20)	Germany	21 ~ 62	(20/20)	8 weeks, 3 times/week, 30 min/time	CBAAD	The CBAAD is a VR game that assesses four attentional functions through daily - life - scenario tasks like visiting a supermarket, driving a car, watching sports, and listening to music while driving.
Manuli et al. (34)	2020	90(49.41)	Italy	11 ~ 43	(30/30/30)	8 weeks, 5 times/week, 60 min/time	MoCA	The Lokomat—Pro’s VR—based feedback module projects exercise results on a flat screen, and the exercises involve patients collecting and/or avoiding randomly distributed objects in the virtual environment to enhance motivation.
Wilson et al. (35)	2021	17(12/5)	Australia	<18	(10/7)	8 weeks, 3 ~ 4 times/week, 30 min/time	MoCA	The EDNA - 22 system’s VR tasks include four goal - based tasks like moving objects to cued or random targets and avoiding distractors, and three exploratory tasks such as creating audio - visual effects by manipulating objects on a touchscreen, all requiring single—or bimanual movements.
De Luca et al. (36)	2022	30(14/16)	Italy	50.5 ± 17.9	(15/15)	8 weeks, 3 times/week, 45 min/time	MoCA	The VRRS used in the study is a rehabilitation system with a magnetic kinematic acquisition system. It offers 2D and 3D exercises for attention rehabilitation, including oculo—motor coordination tasks, pick—and—place, ordering, selection and sequential selection tasks, with augmented feedback to train different attention sub-domains.
Calabrò et al. (37)	2023	40(23/17)	Italy	18 ~ 75	(20/20)	12 weeks, 5 times/week, 60 min/time	MoCA	The VRRS-HomeKit is a tablet with sensors like K-Wand and K-sensors. It enables non-immersive virtual exercises for motor, cognitive and speech therapy, where patients interact with 2D scenarios via touch screen or sensors, with support from a therapist and caregiver during 1-h sessions.
De Luca et al. (38)	2019	100(56/44)	Italy	39.93 ± 10.1	(50/50)	8 weeks, 7 times/week, 60 min/time	MoCA	BTs-N is a semi—immersive VR program where patients interact with virtual scenarios and stimuli via movement on an interactive screen, performing tasks like moving/manipulating objects, making associations, and doing calculations. They select/explore elements in the virtual environment to get feedback, and the difficulty level rises with more distractors and less execution time.
Gangemi et al. (39)	2023	30(19/11)	Italy	18 ~ 65	(15/15)	VRRS Virtual Cognitive Task	EEG	The VRRS cognitive module has interactive activities for cognitive domains like attention and memory. It includes 2D exercises via touch screen or sensor and 3D exercises with wearable sensors, and tasks have three difficulty levels with controlled stimuli and distractors.
Välimäki et al. (40)	2018	90(45/45)	Finland	18 ~ 65	(29/29/32)	8 weeks, 7 times/week, 30 min/time	Simon	The rehab group played CogniFit web—based games, picking one from three cognitive—training categories daily. The entertainment group chose a commercial game from 8 options on PS3.
Pennington et al. (18)	2022	30	U.S	52.8 ± 11.0	(15/15)	9 times in 3 weeks,30 min/time	Scaled score	The VR game used in the article is GoWings Safari by Blue Goji. In this path—based adventure game, players pedal a stationary bike to explore a wildlife environment, scan and target animals or “poachers,” collect rewards, and the faster they pedal, the better they can move, with each session randomly generated.
Rogers et al. (41)	2019	21(9/12)	Australia	42–94	(11/10)	4 weeks, 3 times/week, 30 ~ 40 min/time	MoCA	The VR tasks in the Elements system include goal—directed tasks like Bases, Random Bases, Chase Task, and Go/No-Go, which require moving a hand—held object to targets and resisting distractors, and exploratory tasks such as Mixer, Squiggles, and Swarm, where participants create shapes, sounds, and explore audiovisual relationships, with real-time augmented feedback during the process.

The duration of each intervention session varied between 30 and 60 min, with the majority of sessions lasting approximately 30 min. This suggests a common preference for relatively brief, focused training periods. Regarding the frequency of interventions, the number of sessions varied widely, ranging from a minimum of 4 to a maximum of 12 sessions, indicating the use of different implementation strategies across the studies included in the analysis.

### 3.3 Methodological assessment of the included literature

**Figure 3:**
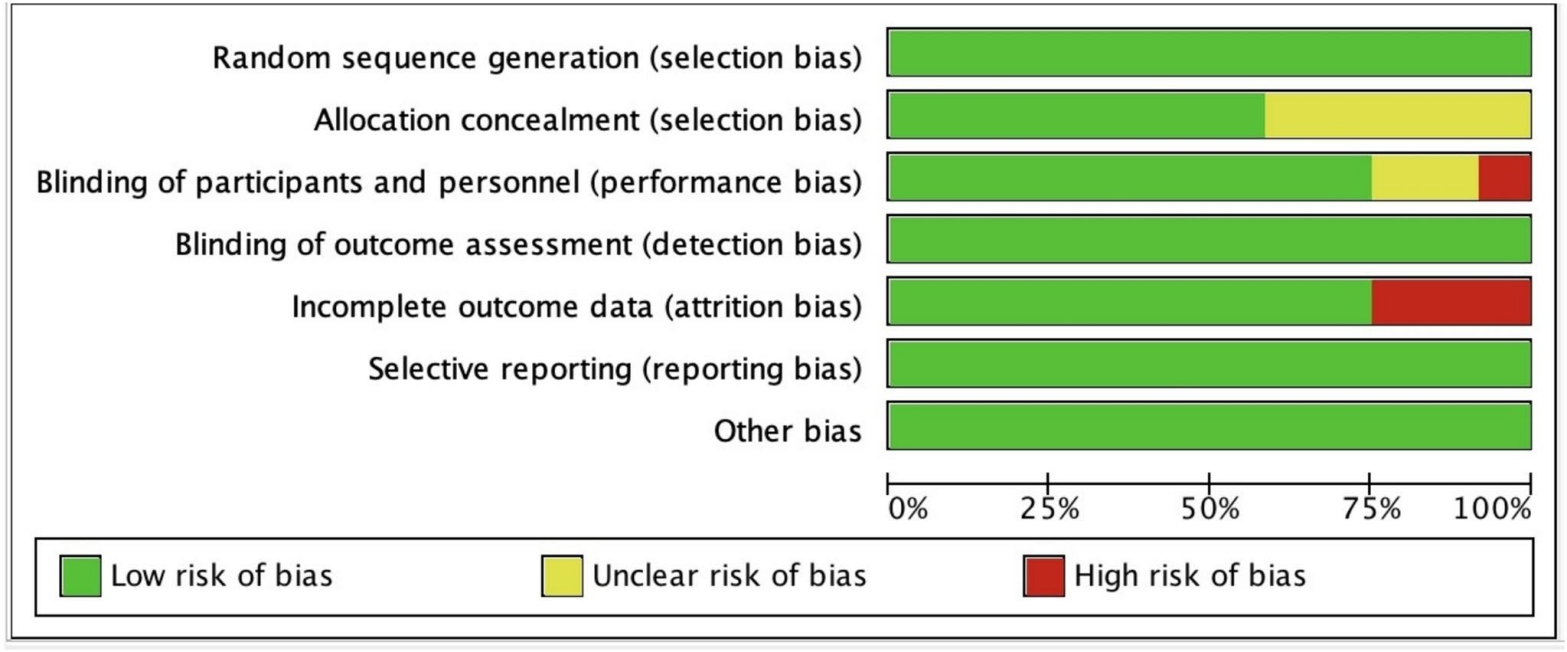
Risk of bias graph.

**Figure 4:**
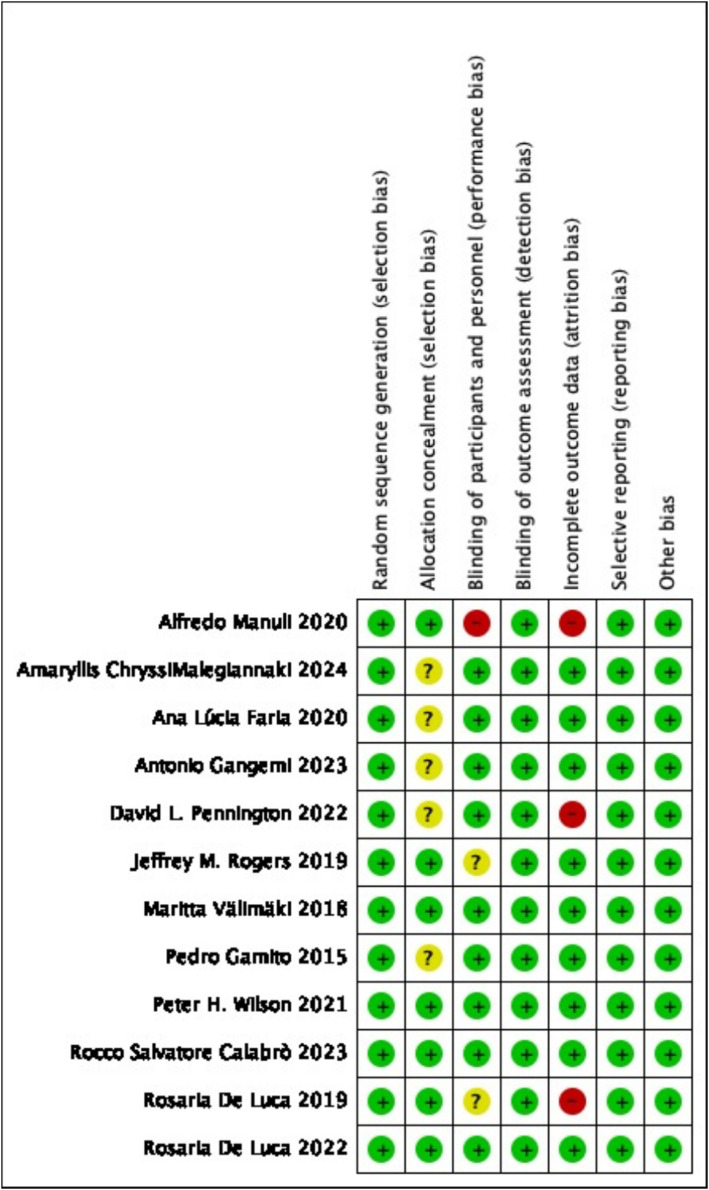
Risk of bias summary.

Table 2 presents the quality ratings of the 12 studies according to the Cochrane Handbook for Systematic Reviews of Interventions. These scores provide a systematic assessment of the methodological rigor and overall quality of the studies. Based on the established criteria for assessing study quality, 4 of the 12 studies were categorized as high-quality literature, indicating that the studies followed sound research methodology and had a high degree of reliability. Five studies were considered to be of moderate quality. A further three studies were rated as low quality literature. The results of the assessment are presented visually in Figures 3, 4, illustrating the distribution of quality scores of the included studies. These charts help to understand the overall quality of the studies and provide a quick visual comparison of the studies.

**Table 2:** Evaluation results of literature quality risk bias of included studies.

Inclusion of literature (author)	Random sequence generation	Allocation concealment	Blinding of participants and personnel	Blinding of outcome assessment	Incomplete outcome data	Selective reporting	Other bias
Manuli et al. (34)	L	L	H	L	H	L	L
Malegiannaki et al. (33)	L	U	L	L	L	L	L
Faria et al. (31)	L	U	L	L	L	L	L
Gangemi et al. (39)	L	U	L	L	L	L	L
Pennington et al. (18)	L	U	L	L	H	L	L
Rogers et al. (41)	L	L	U	L	L	L	L
Välimäki et al. (40)	L	L	L	L	L	L	L
Gamito et al. (32)	L	U	L	L	L	L	L
Wilson et al. (35)	L	L	L	L	L	L	L
Calabrò et al. (37)	L	L	L	L	L	L	L
De Luca et al. (38)	L	L	U	L	H	L	L
De Luca et al. (36)	L	L	L	L	L	L	L

L, Low risk of bias; U, Unclear risk of bias; H, High risk of bias.

### 3.4 Analysis of outcomes

#### 3.4.1 The effect of virtual reality exercise technology on cognitive function in brain-injured patients

**Figure 5:**
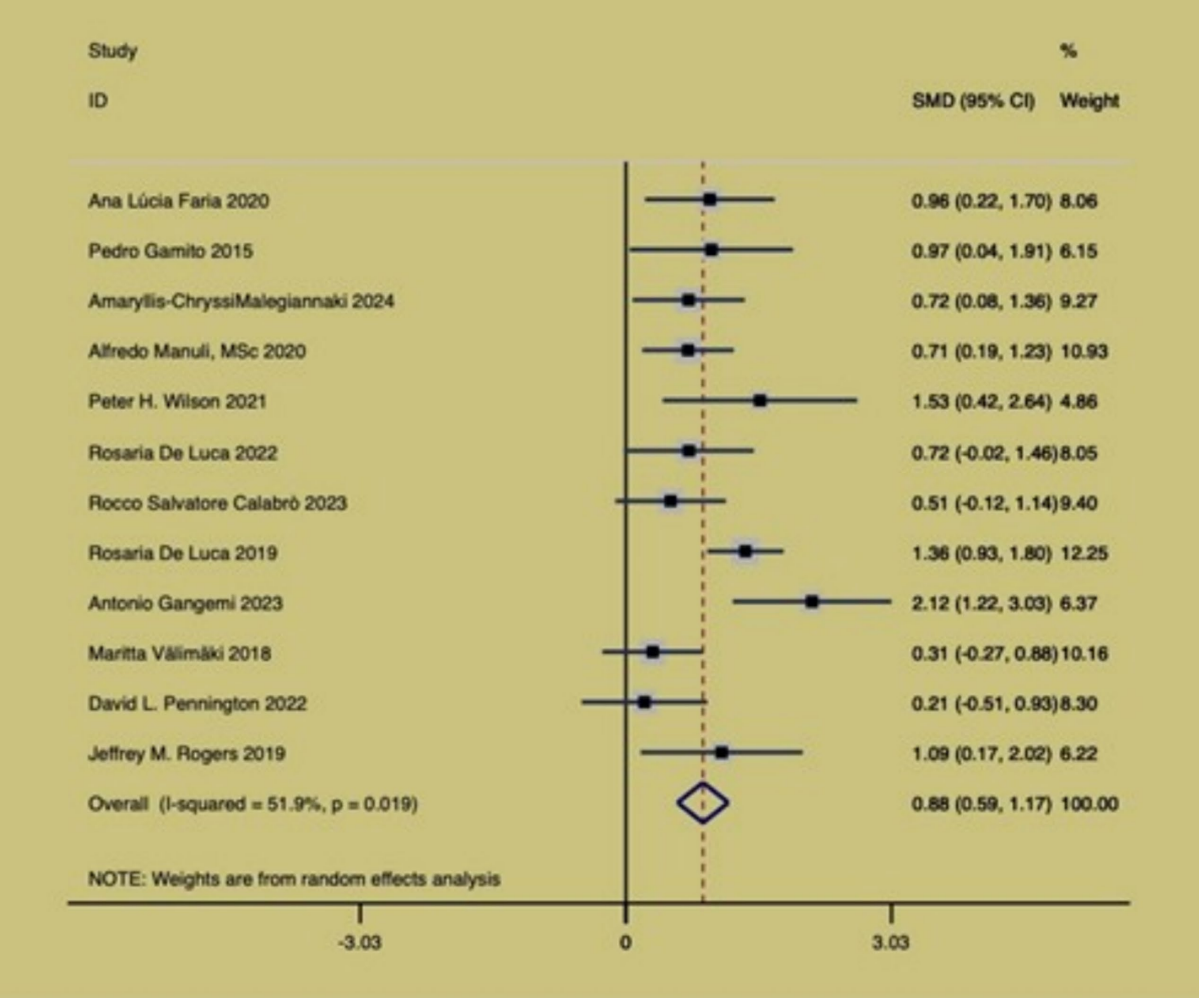
Forest map of key indicators.

Figure 5 shows the effect of virtual reality exercise technology on cognitive function in brain-injured patients. The article included 12 RCT studies, which found that (SMD = 0.88, 95% CI (0.59, 1.17), *p* = 0.019, I2 = 51.9%), moderate heterogeneity of the overall effect, which was statistically significant, and a random effects model was used to analyze the effect size. The results of the study suggest that virtual reality exercise technology has an improvement effect on cognitive function in brain-injured patients. In conducting this meta-analysis, we adhered to rigorous methodological standards to ensure the homogeneity of outcome measures among the included studies, thereby safeguarding the scientific validity and reliability of the analysis results. However, during the process of screening and including studies, we observed that different studies utilized various tools to assess cognition, with a significant number employing the MoCA as an outcome measure. This variation added a layer of complexity to our analysis. To address this, we standardized the data by calculating statistical metrics such as SMD, transforming the cognitive scores from different studies into comparable effect sizes. This approach mitigated the impact of differences in measurement tools, allowing data from various studies to be compared and analyzed within a unified framework.

#### 3.4.2 Sensitivity analysis

**Figure 6:**
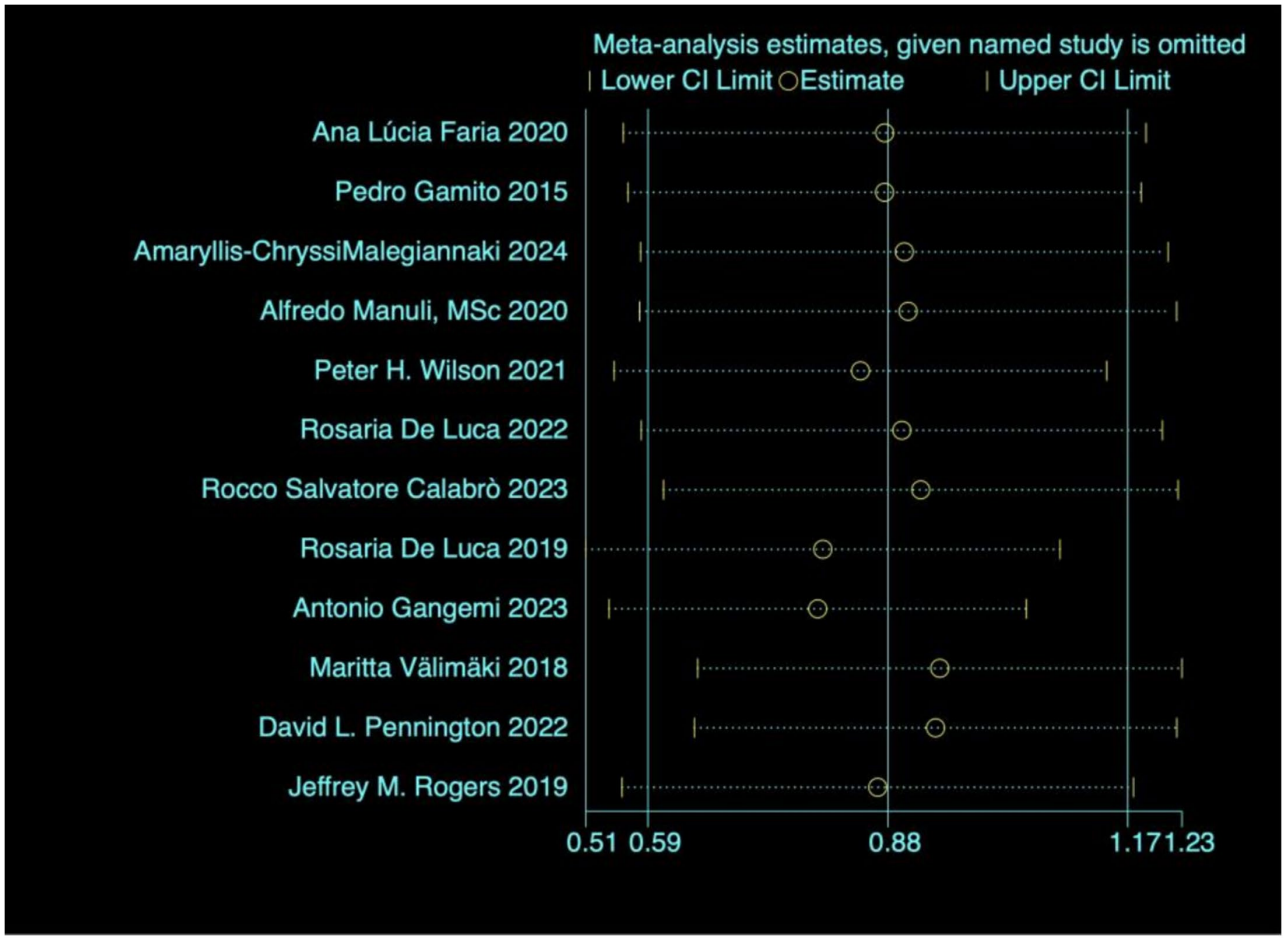
Heterogeneity test.

In order to explore whether the heterogeneity of the studies was caused by a single study, a sensitivity analysis was therefore carried out on the main outcome indicators, as shown in Figure 6, the results were all within 95% confidence intervals and the results were robust enough not to require the deletion of any literature.

**Figure 7:**
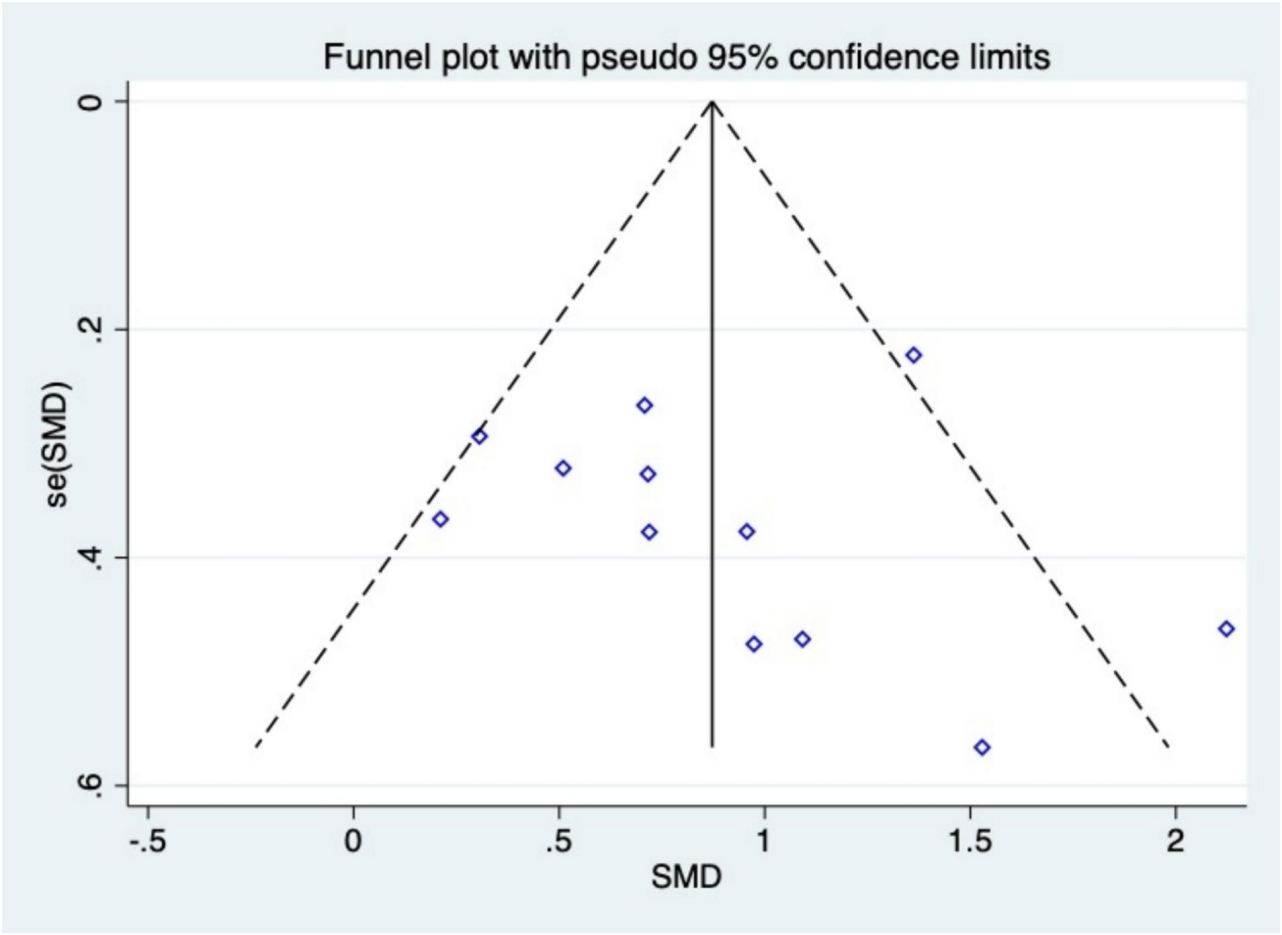
Funnel plot.

The data of studies on the main outcome variables were included, and the funnel plot was used for publication bias test. The results show Figure 7 that the funnel plot is basically symmetrical, so it can be judged that there is no publication bias in the literature.

## 4 Discussion

The effectiveness of virtual reality sports games can be attributed to their unique mechanisms. First, virtual reality technology can effectively motivate and interest patients to participate by creating an immersive and interactive environment. The advantage of this technology lies in its ability to simulate real-world sports scenarios, allowing patients to participate in various exercise training sessions in a safe environment. The VR system achieves multimodal sensory integration through the construction of a three-dimensional visual field, spatial sound engine, and haptic feedback devices. This immersive environment can activate the synchronous neural network activity in the prefrontal cortex, parietal association area, and basal ganglia, providing immediate feedback and multisensory stimulation in a virtual reality setting. This enhances learning outcomes, promotes neuroplasticity, and improves cognitive performance. Additionally, the built-in real-time biofeedback mechanism forms a closed-loop neural remodeling circuit, effectively promoting synaptic plasticity in the cortico-striatal pathway by enhancing the match between motor intentions and actual neural signals. Personalized settings for virtual reality sports games enable therapists to tailor experiences based on patients ‘specific needs and capabilities. This flexibility ensures that treatment plans better align with patients’ actual conditions, contributing to improved rehabilitation outcomes (26). The results of this study indicate that virtual reality exercise game technology has a significant effect in improving cognitive function in patients with brain injury. Specifically, the analysis of the 12 randomized controlled trials included showed SMD = 0.88, 95% CI (0.59, 1.17), *p* = 0.019, and I^2^ = 51.9%, suggesting a statistically significant effect of the intervention of virtual reality motion technology. This finding is similar to the study by Chanpimol et al., who found that virtual reality intervention significantly improved cognitive function in stroke patients (27, 28). The consistency of research findings indicates the reliability of virtual reality intervention in improving the cognitive function of brain-injured patients. VR intervention may enhance cognitive recovery by stimulating specific neural pathways in the brain. Future research could focus on optimizing this intervention, such as exploring more effective VR game designs and determining the optimal intervention duration and frequency. However, the study by Bonanno et al. and others emphasized that individual differences may affect the intervention effect, which is in line with the moderate heterogeneity we observed (I^2^ = 52.1%), suggesting that individual patient characteristics need to be taken into account when applying virtual reality technology (29). Differences in intensity, frequency, and VR content exist among studies, with some using high-intensity daily sessions and more complex games, while others opt for low-intensity weekly ones. Such protocol variations lead to inconsistent results, underscoring the importance of considering patient-specific traits in VR application.

The relevance of this study lies in the fact that with the rising incidence of brain injuries, traditional rehabilitation methods face low participation and limited effectiveness (30). The introduction of virtual reality motor game technology provides strong support for its application in the recovery of cognitive function in brain-injured patients, showing its potential as a novel intervention. By enhancing patients’ physical coordination and reaction speed, virtual reality sports games not only improve their motor abilities, but also further enhance their motivation and participation in learning. Virtual reality sports games can also enhance patients’ rehabilitation through social interaction. In the virtual environment, patients can interact with other participants, and this social element helps to improve patients’ emotional state and mental health. Social interaction can stimulate positive emotions and enhance patients’ sense of engagement, thereby improving rehabilitation.

In this study, heterogeneity exists, affecting result consistency and interpretation. A major source is the non-uniformity of cognitive measurement tools. Different studies use diverse tools with variances in dimensions, assessment, and sensitivity, and examiner scoring may also vary. Such diversity causes data-collection biases, heightening result heterogeneity. Another key source is the complexity of brain-injury patients. Their injury sites, severities, causes, and illness durations differ, and distinct sites impact different cognitive areas. These individual differences in patients significantly increase sample heterogeneity and challenge result consistency. Given the observed heterogeneity, personalized approaches considering patient-specific factors like injury type and severity are needed. Technological optimization, such as developing standardized VR platforms, and exploring combinations with non-invasive brain stimulation, could enhance effectiveness. Long-term follow-up studies are essential to assess the durability of effects, and economic evaluations will aid in clinical adoption. Finally, uncovering the underlying neural mechanisms using advanced neuroimaging will deepen our understanding.

## 5 Conclusion

In conclusion, our study reveals that virtual reality (VR) sports games have significant potential in rehabilitating brain-injured patients. VR sports games effectively enhance patients’ cognitive function, coordination, and reaction speed. These improvements not only benefit daily living but also boost learning motivation and engagement. As an application, VR sports games offer a novel and effective alternative to traditional rehabilitation. They provide an immersive experience tailored to individual needs, making rehabilitation more enjoyable.

Looking forward, future research should explore long-term effects, optimize VR game design for different brain injuries, and better integrate VR into existing rehabilitation programs. With further study, VR sports games will likely play a more crucial role in improving the lives of brain-injured patients.

## Data Availability

The original contributions presented in the study are included in the article/supplementary material, further inquiries can be directed to the corresponding authors.
